# Comparison of Clinical Success of Applying a Kind of Fissure Sealant on the Lower Permanent Molar Teeth in Dry and Wet Conditions

**Published:** 2015-09

**Authors:** Tahereh Eskandarian, Saeid Baghi, Abbas Alipoor

**Affiliations:** 1 Dept. of Pediatric Dentistry, School of Dentistry, Shiraz University of Medical Sciences, Shiraz, Iran.; 2 Pediatric Dentist, Boushehr, Iran.; 3 Student Research Center, Shiraz University of Medical Sciences; Assistant Professor, Mazandaran University of Medical Sciences, Sari, Iran

**Keywords:** Fissure Sealant, Clinical Success, Saliva Contamination

## Abstract

**Statement of the Problem:**

Fissure sealant therapy is among the most effective methods of preventing dental caries. However, it is lengthy and isolation of the teeth is difficult in this procedure especially in young children. Using new hydrophilic fissure sealant may reduce such problems.

**Purpose:**

This study aimed to evaluate the clinical success of a hydrophilic fissure sealant on the lower permanent molar teeth in dry and wet conditions.

**Materials and Method:**

This clinical trial assessed 31 patients (mean age 8.13±1.77 years) who needed fissure sealant therapy on their first or second mandibular permanent molar. Having performed dental prophylaxis, the teeth were etched and rinsed. Then one of the two was randomly selected and sealed with smartseal & loc in isolated and dry conditions; while, the other was wetted on the etched enamel by using a saliva-contaminated micro brush, and was then sealed with the same fissure as the first tooth. Six and 12 months later, two independent observers examined the clinical success of sealant through checking the marginal integrity, marginal discoloration, and anatomical form. Data were analyzed by using SPSS software, version 16. The bivariate Chi-square and Exact Fisher tests were used to compare the clinical success of the two treatment methods.

**Results:**

There was a high interpersonal reliability between the two examiners (K= 0.713). After 12 months, 90.3% clinical success was observed in dry conditions and 83.9% in wet conditions for smartseal & loc; however, the difference was not statistically significant (*p*= 0.0707).

**Conclusion:**

According to the results of this study, it seems that using new hydrophilic fissure sealant can reduce technical sensitivities and consequently decreases the apprehensions on saliva contamination of etched enamel during treatment procedures.

## Introduction

Over the two past decades, developments in dentistry, especially introduction and application of fluoride in the form of toothpastes, mouthwashes, and topical fluoride therapy, as well as fluoridation of drinking water have significantly cut down on tooth decay, particularly on proximal and smooth surfaces of the teeth. Occlusal surfaces of first and second permanent molar teeth are susceptible to decay in young patients and also have lower fluoride absorbing in comparison with smooth surfaces. Although occlusal surfaces constitute only 13% of the whole teeth surfaces, 88% of decays occur on this site.[1-2] Deep grooves on the occlusal surface are the susceptible sites for bacterial products accumulation such that they are impossible to be completely eliminated. Thus, they are very likely to help progress the initial caries rapidly. Since fluoride therapy was effective on the smooth surfaces, many efforts have been made to provide a physical barrier in form of fissure sealant to separate the occlusal surface from mouth environment and subsequently prevent tooth caries to some extent.[3-4] Fissure sealant was first introduced in the mid-1960s in form of some materials derived from cyanoacrylates family. However, due to the gradual demolition of these compositions by bacteria, they were restricted to be used only in experimental trials and studies.[5] With the introduction of acidic-etched method in 1969 and evolution of resin fissure sealant, these materials acquired special place in dentistry.[3-5] By 1971, the first fissure sealant with resin base was marketed by NUVASEAL trademark.[5]

Before fissure sealant treatment, etching is performed to make the porosities appear on the enamel and increase the surface energy. During this time, acrylic resin fluid without filler or with micro-filler and low viscosity is applied on the enamel. The resin penetrates into micro pores which are produced by etching to form resin tags[5-6] which in turn, leads to a strong bond between the resin and etched enamel. Finally, resin is applied to cover all grooves which are susceptible to carries and also to prevent penetration of debris, bacteria, and so on.[1] The clinical success of any kind of pit and fissure sealants is directly linked to their ability to remain bonded to the occlusal pits and fissures.[7]

One of the most important reasons of failure of fissure sealant treatment is contamination of enamel surface with saliva.[8-9] In order to reduce the sensitivity to saliva and moisture during treatment, application of hydrophilic dentin bonding material was introduced in 1992.[10] Thereafter, numerous studies reported that application of hydrophilic bonding material increased the fissure sealant retention in case of contamination with saliva.[8-11]

The studies by Perdiago *et al.*,[8] Hebling and Feigal,[9] and Hit and Feigal[10] showed that bonding agent increased the bond strength when applied under the fissure sealant. Askarizadeh *et al.*,[11] in addition to Asselin *et al.*,[12] in 2008 revealed that applying a layer of hydrophilic bonding on an etched teeth contaminated with saliva would decrease the rate of fissure sealant microleakage.

When the etched enamel is contaminated with saliva, saliva that contains protein would diffuse into the pores on the basis of osmotic rule. It decreases the retention and increases the microleakage of fissure sealant; so this diffusion needs to be inhibited. Heydari *et al.* demonstrated that any contact time less than 5 seconds between the saliva and etched-enamel would not help diffuse saliva composition and thus would not produce any significant difference in retention and microleakage.[13]

There are still controversies over using the conventional methods or the newer methods such as self-etched bonding material. Chasqeira *et al.*,[14] in their* in vivo,* study concluded that the self-etching adhesive systems (Prompt-L-Pop and Xeno III) produced similar satisfactory shear bond strength values between fissure sealant material and superficial enamel. Highly strong self-etched systems would produce strength equal to acid-etch and rinse methods. However, their highly acidic pH (pH≤1) significantly weakens the bond strength. Moreover, the retention is merely mechanical in this method and acid-etch and rinse method seems to be the best and most effective method to achieve suitable adhesion to teeth surfaces.[14-15]

Based on the evidence-based studies, the American Dental Association (ADA) Council on Scientific Affairs announced that self-etch bonding materials that do not include separate stages for etching procedure are not suggested due to their lower adhesion compared to the conventional methods.[16-17] Contrary to the old fissure sealants, the new generation of fissure sealants promoted under the name of modern-fissure sealant have hydrophilic characteristic that eliminate the need for bonding material. Hence, the clinical sensitivity of fissure sealant treatment would be decreased, the number of stages gets reduced, and a rapid treatment s would be achieved. Compared with the conventional fissure sealant, these sealants have 50% more filler; nonetheless, the filler particles are nano-sized (<1μm) which helps them have high resistance to friction.[18]

Blesch’s study (2007) represented that using a kind of modern hydrophilic fissure sealant would eliminate the need for bonding application even in case of enamel contamination with saliva. Additionally, it had not seen any change in fissure sealant margin or fissure sealant superficial quality; in fact the need for bonding was purged after etching.[18]

The present study assessed a kind of hydrophilic fissure sealant in two conditions, dry and wetted with saliva. This fissure sealant is a new generation of composite-based fissure sealant and, based on the manufacturer’s claims, its hydrophilicity makes it applicable in wet condition in spite of absence of suitable isolation in children. On the other hand, wiping out the saliva would be difficult in children; thus, the objectives of this study were focused on the clinical success of hydrophilic fissure sealant in the above-mentioned conditions. 

## Materials and Method

In this clinical trial study, 31 patients aged 6-12 years old were selected from those referring to the Department of Pediatric Dentistry at Shiraz Dental School. The patients had at least a permanent molar tooth on either side of the mandible which was completely erupted. Inclusion criteria for participation in this study were having completely erupted first or second molar mandibular teeth, presence of complicated and deep grooves on the teeth surfaces, absence of occlusal or interproximal caries, being categorized as a low risk patients based on the caries and oral hygiene, and approving the participation and filling the consent form to cooperate with the researchers during the trial periods .As long as the ethics was concerned, the patients were summoned for re- treatment in case of failure of fissure sealant therapy after the end of the study.

The follow-up period was decided to be 6 months with respect to the fissure sealant guidelines and the previous studies.[17-19] After selecting the patients, one of the two mandibular permanent molars was randomly selected to be treated with method A, and the other was left to be treated with method B.

In method A, the occlusal surface was rinsed with brush and hand piece (low speed) and after being isolated by cotton rolls, the tooth was etched with 35% phosphoric acid (Ultradent; USA) for almost 20 seconds and then rinsed.

The isolation by cotton rolls was adopted respecting the results of previously performed investigations, as well as the pediatric dentistry reference texts that had mentioned performing fissure sealant does not require isolating the teeth by rubber dam, and that isolation by cotton rolls can yield the satisfactory results.[3-4,20] After etching, the related fissure sealant material (smartseal & loc; Detex, Germany) was put on the tooth by using an explorer to avoid any possible bubbles. According to the manufacturer’s guideline, the tooth was light-cured for 20 seconds. The fissure sealant was re-examined for not having bubbles and occlusal interruption, and complete marginal integrity.

In method B, the other tooth was isolated by using cotton rolls. The tooth surface was rinsed and dried by using a micro brush. Similar to method A, the tooth was etched, rinsed and dried. Then, a saliva contaminated micro brush was used to wet the etched enamel. The tooth was dried so that the sealant could be placed on it. Light-curing was done as in method A. Having assessed the tooth, all cotton rolls were then removed.

The patients were summoned 6 and 12 months later. Two independent blind observers examined the teeth clinically using Feigal’s scores (Table 1).[1] Based on this method, the fissure sealant was considered to be successful when the code of marginal integrity and marginal discoloration was 0 or 1; and with respect to the anatomical form, codes 2a or 7 indicated success of fissure sealant. Other codes meant failure in treatment. Statistical analysis was separately done in 6 and 12 months. Uni- variate descriptions with percentage based values were studied for categorizing. Bivariate analysis including Chi-Square and Fisher Exact tests was used to compare the success of the two fissure sealant treatment methods in dry and wet conditions. Inter-observer agreement was computed by Kappa coefficient. Statistical analysis with determining two-sided p-value was done by using SPSS software, version 16. Significant level was set at *p*˂ 0.05.

**Table 1:** Feigal’s table for clinical success of fissure sealant on occlusal surface

**Rating**	**Assessment Type**
A. Marginal Integrity	
0	Restorative material adjacent to the tooth and not detectable with an explorer
1	Margin detectable with the explorer
2	Crevice along the margin of visible width and depth
3	Crevice formation with exposure of central fissure
B. Marginal Discoloration	
0	No color change at the tooth-sealant interface
1	Discoloration noted along the margin in one area
2	Discoloration noted along the margin in multiple areas
3	Severe discoloration with evidence of penetration and leakage
C. Anatomical Form	
0	Harmonious and continuous with occlusal form and structure
1	Change in anatomical form but all pits and fissures covered
2a	Loss of sealant from one or two pits or accessory grooves (partial loss), but no need to repair or replace sealant
2b	Loss of sealant from pits or accessory grooves (partial loss), with a need for replacement or repair of the sealant
3	Loss of sealant from all pits (total loss)
7	Partial loss due to occlusion
9	Bubble (not connected with the margins)

## Results

The present clinical trial study assessed 31 patients (62 teeth), aged 6 to 12 years (Mean±SD age 8.13±1.77). 

The inter-observer agreement test (K=0.713, *p*= 0.0001) showed that there was an agreement between the two observers.

As represented in Table 2, the rate of success after 6 months was equal in both dry and wet conditions; so there was not any significant difference between them. The results obtained from two of the investigated domains including marginal integrity and marginal discoloration were completely similar (*p*= 1). In anatomical form, one item was rated higher in wet condition rather than the dry condition; the difference was not statistically significant, though (*p*= 1).

**Table 2:** Fissure sealant treatment success rate after 6 months period in dry and wet conditions

**Domain**	**Fissure sealant ** **treatment condition**		P.value*
	**Wet**	**Dry**	
Marginal Integrity	30(96.8)	30(96.8)	1
Marginal Discoloration	31(100)	31(100)	1
Anatomical Form	30(96.8)	31(100)	1
Net Success	30(96.8)	30(96.8)	1

*Two-sided Fisher Exact test

Table 3 displays the result obtained after 12 months. Both methods were similar in marginal discoloration (*p*= 1) but in terms of marginal integrity, application of fissure sealant in dry condition had rather better results; however, the difference was not statistically significant (*p*= 1). Regarding the anatomical form, at the end of 12 months, the success rate in dry condition was also higher than the wet condition; yet, the difference was not statistically significant (*p*= 0.671). Generally, the success rate of fissure sealant application was 90.3% in dry condition and 83.9% in wet condition; however, the two method did not differ significantly (*p*= 0.707).

**Table 3:** Fissure sealant treatment success rate after 12 months period in dry and wet conditions

**Domain**	**Fissure sealant ** **treatment condition**		P.value*
	**Wet**	**Dry**	
Marginal Integrity	30(96.8)	29(93.5)	1
Marginal Discoloration	31(100)	31(100)	1
Anatomical Form	29(93.5)	27(87.1)	0. 671
Net Success	28(90.3)	26(83.9)	0.707

*Two-sided Fisher Exact test

## Discussion

One of the most important factors in fissure sealant failure is contamination of etched enamel surface with saliva.[8-9] In young children and mentally retarded patients, it is very challenging to control salivary flow. Older studies had mentioned that shorter time of contact between etched enamel surface and saliva could help preventing resin fluid penetration into microporosities. Likewise, recent studies represented that diffusion of saliva material into the etched enamel pores depends on time; i.e. restricting the time of contact between saliva and etched-enamel surface to less than 5 seconds would leave no considerable negative effect either on the retention factor or on fissure sealant microleakage.[13, 20] The use of dentine bonding agents containing acidic hydrophilic monomers as a layer between the etched enamel and fissure sealant was first reported in 1992. It was found to be able to increase the fissure sealant retention and decrease the microleakage when enamel is contaminated with saliva. This was because the traditional and conventional fissure sealant could not tolerate even a little moisture due to their hydrophilic feature.[13] To solve this problem, Erdemir *et al.* conducted an *in vivo* study[21-22] and used flowable composites combined with a total-etch adhesive to seal pits and fissures; however, this method seemed to be as sensitive as using hydrophilic bonding agents with pit and fissure sealants.

Fissure sealant is commonly used in the age range, during which the patient’s cooperation is weak and tooth isolation is difficult. So, fissure sealant therapy is suggested to be applied using a rapid easy-to-use method with less technical sensitivity.[5] Introduction of new generation of self-etched adhesive bonding has improved working stages and reduced treatment technical sensitivity. Nevertheless, concerns still exist about the insufficient adhesion between the restoring material and teeth, which happens as a result of contamination with saliva and bacteria through the gaps which causes recurrent caries and pulp diseases. Applying adhesive material reduced the microleakage but did not resolve it completely. Numerous studies evaluated the clinical success rate of hydrophilic bonding material such as self-etch for bonding the fissure sealant to teeth. For instance, in the 3-year clinical trial enrolled by Munoz *et al.*, the success rate was reported to be 95%.[23] In another study by Baghosion[24] and Manhart *et al.*,[25] the success rate was reported to be 96%; although in the study by Backett *et al.*, (2002) 23% failure was proclaimed after one year.[26]

These studies did not use any standard criteria regarding bonding in clinical research and studies, so comparing the results would be difficult. Peumans and Kanumilli[27] in their study about the clinical effectiveness of dental adhesive material concluded that different results contribute to the weak connective characteristic of self-etch material in comparison with conventional rinse and acid-etch method. Insufficient thickness of hybrid layer, separated hydrophilic and hydrophobic phases of compositions, and hydrolysis sensitivity led to increasing treatment technical sensitivity. Acid-etch and rinse method was approved by most studies as the best and most effective way to achieve high adhesion to teeth.[7-22]

Concerning the numerous reported cases of self-etch bonding materials, it seems that it would be better to use the conventional etch-and-rinse method and apply new hydrophilic fissure sealant to preserve adhesion properties in the presence of diminutive moistures. Applying self-etch bonding material as adhesive agent of fissure sealant material to teeth is a conservative method and should be done with regular patient follow ups;[28] because compared with etch-and-rinse method, self-etch materials have lower adhesive characteristics.[17] It is attributed to the fact that these materials produce hybrid layer of higher quality, longer resin outgrowths, as well as creating better and regular etching pattern in conventional acid-etch and rinse method, which is another stronger success reason assigned for the present study.

In the present study, the success rate was 90.3% in dry condition and 83.9% in wet condition, but the statistical analysis did not demonstrate any significant differences (*p*= 0.0707). It might have been because the long duration of contact between the teeth and saliva affected its influence on the enamel porosities; i.e. reducing it to less than 5 seconds would prevent it from negatively affecting the fissure sealant retention.[13]

In comparison with other studies which used conventional etch-and-rinse method, this study represented the same or better results (83.9% in this study versus 61% in Feigal’s study).[9] Furthermore, compared with other studies which applied self-etch bonding;[23-24] the present study achieved better or rather similar results. The reason was the acidic-hydrophilic monomer in these types of fissure sealants; whereas, the conventional fissure sealant present in the market were hydrophobic such that they did not influence the etched enamel if they got wet. On the other hand, in a study performed by Karami *et al.*[29] the clinical effectiveness of fifth and sixth generations of bonding material on contaminated enamel fissure sealant were compared and the success rate of using hydrophilic bonding after 12 months was 66.7% in contaminated condition and 53.3% in isolated condition.

The results of the present study are probably more accurate because we employed three separate stages of acid etch-and-rinse, application of bonding material, and inserting the fissure sealant. Mascarenhas *et al.*[30] demonstrated that using hydrophilic bonding under the fissure sealant achieved 64% success rate; it was in contrast with the results of the present study which found 83.9% success rate. The difference can be attributed to the duration of follow-up which was 2 years in their study and 1 year in the current one; also different assessment criteria were applied.

Lyqidakis *et al.* enrolled a study with 4-year follow up.[31] They demonstrated 70.2% success rate for fissure sealant when using hydrophilic bonding material; while, without using bonding material, the success rate declined to 25.5% with complete retention, and 44.6% with partial loss. The result of the above-mentioned study was weaker than the current one, which can be because of the 4-year follow-up and the different clinical criteria that assessed the fissure sealant success rate. The findings of the present study were compatible with Blesch's 6-month study on application of smartseal & loc fissure sealant.[18]

It seems that applying new generation of fissure sealant containing hydrophilic monomers that tolerates more moisture accompanied by etch-and-rinse method that provides the best conditions for fissure sealant, could reduce the technical sensitivities during treatment. Additionally, in case of contamination with saliva, the need for a hydrophilic bonding material is resolved. Consequently, with the reduced treatment time, the cooperation of patients was increased. 

## Conclusion

Although in fissure sealant treatment, complete isolation and dry conditions are suggested, the results of this study demonstrated that using new hydrophilic fissure sealant that contains hydrophilic monomers such as smartseal & loc due to their tolerance of moistures can reduce the technical sensitivities as well as the concerns about saliva contamination of etched enamel during the treatment. Besides, unlike the previous studies, applying fissure sealant requires hydrophilic bonding material under the fissure sealant to decrease the treatment time, and thus increase cooperation and better patient admission. 
